# Club Drugs and HIV/STD Infection: An Exploratory Analysis among Men Who Have Sex with Men in Changsha, China

**DOI:** 10.1371/journal.pone.0126320

**Published:** 2015-05-07

**Authors:** Xi Chen, Xingli Li, Jun Zheng, Junshi Zhao, Jianmei He, Guoqiang Zhang, Xuemin Tang

**Affiliations:** 1 Department of AIDS/STI Control and Prevention, Hunan Provincial Center for Disease Prevention and Control, Changsha, China; 2 School of Public Health, Central South University, Changsha, China; Temple University School of Medicine, UNITED STATES

## Abstract

**Objective:**

To evaluate current club drug use and its potential association with the transmission of HIV/STD among Changsha men who have sex with men (MSM).

**Method:**

A cross-sectional survey was conducted by using self-administered questionnaires including information regarding socio-demographics, club drug use, high-risk behaviors, and HIV/STD infections. Multiple methods including venue-based, peer referral using “snowball” techniques, and internet advertisements were used to recruit study participants.

**Results:**

Of the 826 participants, 177 (21.4%) reported that they had used club drugs at some time before or during sex in the past six months. MSM with young age, low education level, and seeking partners through the internet or bars were the main population who used drugs. Poppers were the most common drug used among Changsha MSM. The prevalence of HIV, syphilis, and herpes simplex virus-2 were higher among drug users. There were no significant differences in unprotected sexual intercourse and condom use between drug users and non-users. Compared with non-users, risk behaviors such as group sex, multiple sex partners, and sex with foreigners were more frequent among drug users.

**Conclusion:**

Club drug use is common among Changsha MSM, and is related to unsafe sex activities and HIV/STD infection. It is necessary to build novel targeted HIV prevention strategies to monitor and reduce club drug use among MSM.

## Introduction

The HIV/AIDS prevalence among men who have sex with men (MSM) in China has increased rapidly in recently years. According to a CDC report, MSM accounted for approximately 12.2% of the newly reported HIV/AIDS cases in 2007[1] and nearly 32.5% in 2009[2].

Early studies suggested that a high proportion of multiple sexual partners[3] and low rates of condom use [4–6] were the main reasons for the rapid increasing HIV prevalence among MSM. Recently, some reports found that a high prevalence of club drug use and related high-risk behaviors were additional possible reasons[7–10]. Some studies in western countries demonstrated that club drug use before and during sex might increase the likelihood of high-risk sexual behaviors[11–13] and facilitate HIV/STD infection[14]. Similar to western countries, China has also seen a dramatic increase in the use of club drugs such as poppers, methamphetamine, ketamine, and ecstasy[15]^.^ These drugs are rapidly replacing heroin and becoming the most widespread illicit drugs in China[16]. These synthetic drugs are used widely in clubs, dance halls, and places where rave parties occur. In 2010, approximately 120,000 (55%) of newly identified drug users used synthetic drugs [16].

Recently, several Chinese studies reported the extent of club drug use among Chinese male commercial sex workers(MCSW), and MSM in some regions. However, the prevalence and pattern of club drugs use varies in different regions and populations [17–18], and studies assessing the relationship between club drug use, sexual risk behaviors and HIV/STD infections are scarce.

In the current study, a cross-sectional investigation in Changsha was conducted to explore the prevalence of club drug use and assess how it relates to the spread of HIV/STI among Chinese MSM. These preliminary findings provide evidence for better implementing targeted interventions measures and strategies for MSM.

## Materials and Methods

### Definition of club drug and poly-drug use

Club drugs were defined as a group of substances with a connection to a club, dance scene, and rave culture [19] such as ketamine, ecstasy/MDMA, GHB, cocaine, and methamphetamine. Poly-drug use was defined as actively combining two or more substances at the same time (i.e., simultaneously or concomitantly)[20,21].

### Study Site

Hunan is a landlocked province located in south-eastern China. The current study was conducted in Changsha (the Capital of Hunan province), which is famous for rich media and entertainment industries. There are hundreds of clubs, dance halls, and karaoke halls in the city.

### Participants

MSM living in Changsha were recruited from August to November 2009 by using multiple methods, including at venue-based locations such as gay bars, gay saunas, parks, peer referral using “snowball” techniques, and internet advertisements. All methods were performed by trained staff at Hunan provincial CDC.

The enrollment criteria for the study were as follows: (1) males aged at least 16 years old (2) Self-report sexual behaviors with a male partner at least once in their lifetime (3) understood the purpose of the study and were willing to participate and sign informed consent.

### Data collection

Self-administered questionnaires were provided and were completed by the study subjects. The questionnaires included several lines of questions as follows. (1) Demographic data including age, race, education, marital status, income, and self-reported sexual orientation. (2) Sexual behaviors, including age at first sexual intercourse with a man, the number of sexual partners, unprotected anal and oral sex in the past six months, and condom use with casual and stable partners. (3) Information regarding drug use including asking whether participants had taken drugs in the past six months. They were also asked two questions: had they ever combined one drug with another (yes/no), and, if yes, which drugs they had combined. (4) Any STD-related symptoms in the past six months.

Blood samples were collected from all eligible participants and used to assess the presence of HIV, syphilis, and herpes simplex virus 2 (HSV-2). Blood specimens were tested for HIV antibodies using enzyme-linked immunosorbent assay (ELISA) and HIV-1/2 western blotting. Syphilis antibodies were detected using a rapid regain test (RPR, Diagnosis t; Shanghai Kehua, China) and were confirmed using a Treponema pallidum particle test (TPPA, Serodia, Japan); participants were determined to be positive for syphilis if both the RPR and TPPA tests were positive. HSV-2 infection was determined by HSV-2 enzyme-linked immunosorbent assay (Herpes Simplex Virus 2 IgG, Institut Virion\Serion GmbH,*Germany*), as per the manufacturer’s instructions.

The study protocol was approved by the Institutional Review Board (IRB) of the First Affiliated Hospital of China Medical University (CMU).

### Statistical analysis

Data were double entered and verified using Epi-Data version 3.0. Data analyses were performed using SPSS for Windows, version 13.0. Continuous variables and categorical variables were presented as means and proportions, respectively. Risk factors were assessed using chi-square statistics for categorical variables. Multiple logistic regression model was used to analyze the risk factors associated with HIV/STD infection and club drug use respectively. A backward procedure was used to select significant variables. Statistical significance was set at 0.05.

## Results

### Characteristics of the study participants

Of the 826 eligible participants, 584(75.0%) were recruited from gay-oriented venues such as bars, night club and tea bars, 168(20.3%) from the internet, and 74(4.7%) from saunas, bath, and park. 177 (21.4%) reported that they had used club drugs at some time before or during sex in the past six months. Of these, 164 drug users were younger than 35 years old (92.6%), 82.5% were single, 27.1% had education of middle school or less, and 62.1% identified themselves as homosexual. Among the 649 non-users, 57.9% identified themselves as bisexual, 80% were aged <35 years, and >90% had a high school or higher level of education. (Table 1).

**Table 1:** Characteristics of the MSM participants who used or did not use club drugs in Changsha, China.

Factors	Drug users (*n* = 177)	Non drug use (*n* = 649)	Total (*n* = 826)	*P-value*
Age (mean,year)	26.7±6.3	28.4±8.5	28.0±8.1	0.006
Age (year)				
<25	78 (44.1)	265 (40.8)	343 (41.5)	0.002
25~35	86 (48.6)	265 (40.8)	351 (42.5)	
≥35	13 (7.3)	119 (18.3)	132 (16.0)	
Marital status				
Single	146 (82.5)	499 (76.9)	645 (78.0)	
Married/cohabitant	27 (15.3)	115 (17.7)	142 (17.2)	0.173
Separated/divorced/widowed	4 (2.2)	35(5.4)	39 (4.8)	
Education				
Middle school or less	48 (27.1)	62 (9.6)	110 (13.3)	0.000
High school or higher	129 (72.9)	587 (90.4)	716 (86.7)	
Income (yuan/month)				
<1000	38 (21.5)	150 (23.1)	188 (22.7)	
1000~	57 (32.2)	252 (38.8)	309 (37.4)	0.123
≥3000	82 (46.3)	247 (38.1)	329 (39.9)	
Sexual orientation				
Homosexual	110 (62.1)	383 (59.0)	493 (59.7)	
Bisexual	44 (24.8)	204 (31.4)	248 (30.0)	0.152
Uncertain	2 3 (13.1)	62 (9.6)	85 (10.3)	
Venues for meeting sex partners				
Internet	153 (86.4)	517 (79.7)	670 (81.1)	
Bathhouses	8 (4.5)	38 (5.9)	46 (5.5)	
Bars and clubs	68 (38.4)	66 (10.2)	134 (16.2)	0.000
Parks and toilet	4 (2.3)	29 (4.45)	33 (4.0)	
Dance halls	10 (5.6)	21 (3.2)	31 (3.7)	

Data are presented as number (%).

### Drug use and risk factors analysis

Popper, compound codeine phosphate oral solution, and ketamine were the commonly used club drugs, especially single popper use, among Changsha MSM. Of the 177 drug users, 153 were single drug users, and 24 were poly-drug users. Of the 153 single drug users, 86.9% (133/153) used popper (volatile nitrite), 5.2% used compound codeine phosphate oral solution (8/153), 3.3% (5/153) used ketamine, 2.6% (4/153) used methamphetamine, and 2.0% (3/153) used ecstasy. Among the poly-drug users, poppers were the most common drugs combined with other agents. No participants reported injecting drugs.

Logistic regression results showed that young (age25–35years vs.age≥35 years, aOR = 3.213, 95%CI:1.628,6.340; age<25 years vs.age≥35years, aOR = 3.629, 95%CI:1.796,7.330), low education level(aOR = 3.671,95%CI:2.277,5.918),seeking sexual partners through the internet(aOR = 1.860, 95%CI:1.101,3.141), seeking sexual partners through the bars (aOR = 4.813, 95%CI:3.175,7.297) were risk factors of club drug use (Table 2).

**Table 2:** Factors associated with club drug use among MSM in Changsha, China.

	β	Wald *x* ^*2*^	P-value	aOR	95%CI	
					lower	upper
Age group						
<25	1.289	12.908	0.000	3.629	1.796	7.330
25~	1.167	11.325	0.001	3.213	1.628	6.340
> = 35(R)						
Education						
Middle school and less	1.300	28.469	0.000	3.671	2.277	5.918
High school(R)						
Seeking partner through the internet						
No(R)						
Yes	0.620	5.379	0.020	1.860	1.101	3.141
Seeking partner through the bars						
No(R)						
Yes	1.571	54.761	0.000	4.813	3.175	7.297
constant	-4.029	59.139	0.000	0.018		

Note: R:reference group

### Sexual risk behaviors

Among the MSM surveyed, more than 40% had unprotected anal intercourse, and >80% had unprotected oral intercourse. There was no significant difference between drug users and non-users. Compared with non-drug users, 4.5% of the 177 drug-using respondents reported having paid sex with men (OR = 2.00, 95%CI: 0.83,4.79), 10.2% reported having group sex (OR = 1.82, 95%CI:1.01,3.27), 2.8% had sex for money (OR = 2.67, 95%CI: 0.84,8.50), 4.5% had sex with a foreign man (OR = 2.51, 95%CI: 1.01,6.25), and 12.4% had sex with women in the past 6 months (OR = 0.82, 95%CI: 0.49,1.34). Fifty-seven percent of drug-using participants and 41.0% of non-drug users reported multiple sex partners (OR_2–4partners vs. 1 partner_ = 1.77 95%CI: 1.26,2.52; OR_>5 partners vs. 1 partner_ = 2.57; 95%CI: 1.29,5.13; Table 3).

**Table 3:** Risky sexual behaviors in MSM who used or did not use club drugs in Changsha, China.

Factors	Drug use (*n* = 177)	Non drug use (*n* = 649)	*p*-value	OR (95%CI)
Unprotected anal intercourse with men (last sexual contact)				
Yes	74 (43.8)	278 (42.8)	0.806	0.96 (0.68,1.34)
No	103 (56.2)	371 (57.2)		
Unprotected oral sex (last sexual contact)				
Yes	158 (89.3)	549 (84.6)	0.117	1.52 (0.90,2.55)
No	19 (10.7)	100 (15.4)		
Paid sex with men in the past 6 months				
Yes	8 (4.5)	15 (2.3)	0.113	2.00 (0.83,4.79)
No	169 (95.5)	634 (97.7)		
Sex for money with men in the past 6 months				
Yes	5 (2.8)	7 (1.0)	0.085	2.67 (0.84,8.50)
No	172 (97.2)	642 (99.0)		
Sex with women in the past 6 months				
Yes	22 (12.4)	96 (14.8)	0.426	0.82 (0.49,1.34)
No	155 (87.6)	553 (85.2)		
Sex with a foreign man				
Yes	8 (4.5)	12 (1.8)	0.04	2.51 (1.01,6.25)
No	169 (95.5)	637 (98.2)		
Group sex in the past 6 months				
Yes	18 (10.2)	38 (5.8)	0.043	1.82 (1.01,3.27)
No	159 (89.8)	611 (95.1)		
Number of male sex partners in the past 6 months				
1	77 (43.5)	382 (58.8)	0.001	
2–4	86 (48.5)	240 (37.0)		1.77 (1.26,2.52)
5+	14 (8.0)	27 (4.2)		2.57 (1.29,5.13)

Data are presented as numbers (%)

The main places that drug users and non-drug users met their sex partners were via the internet (86.4% vs. 79.7%, respectively), bars and clubs (38.4% vs. 10.2%), dance halls (5.6% vs. 3.2%), and bathhouses (4.5% vs. 5.9%).

### HIV/STD prevalence and risk factors analysis

The prevalence of HIV, syphilis, and herpes simple virus 2 infections were higher among drug users compared with non-users. The positive rate of HIV among drug users and non-users was 18.6% and 10.6%, respectively (OR = 1.93; 95%CI: 1.22, 3.03). The prevalence of syphilis was 12.4% (22/177) and 6.0% (39/649), respectively (OR = 2.22; 95%CI: 1.28,3.86). The prevalence of HSV-2 among drug users and non-users was 16.9% (30/177)and 12.8% (83/649), respectively (OR = 1.39; 95%CI: 0.88,2.19) Nineteen point eight percent of drug users and 10.3% of non-users had self-reported STD-related symptoms during their lifetime (OR = 2.14; 95%CI: 1.37, 3.35; Table 4).

**Table 4:** Prevalence of HIV/STIs among drug and non-drug using participants.

Factors	Drug use (*n* = 177)	Non drug use (*n* = 649)	*P*-value	OR (95%CI)
HIV infection				
Yes	33 (18.6)	69 (10.6)	0.004	1.93 (1.22,3.03)
No	144 (81.4)	580 (89.4)		
Syphilis				
Yes	22 (12.4)	39 (6.0)	0.004	2.22 (1.28,3.86)
No	155 (87.6)	610 (94.0)		
HSV-2				
Yes	30 (16.9)	83 (12.8)	0.153	1.39 (0.88,2.19)
No	147 (83.1)	566 (87.2)		
STD-related symptoms				
Yes	35 (19.8)	67 (10.3)	0.001	2.14 (1.37,3.35)
No	142 (80.2)	582 (89.7)		

Data are presented as numbers (%)

Multivariate analysis was used to identify the factors associated with HIV/STD infection by controlling some possible confounding factors. HIV/STD infection (yes/no) was employed as the dependent variable. The participants with any positive results of HIV, Syphilis or HSV-2 test were categorized as HIV/STD positive group, otherwise as HIV/STD negative group. Age, education, unprotected anal sex, unprotected oral sex, group sex, sex with foreigners, number of sex partners, and club drug use were used as the independent variables. Table 5 indicated that HIV/STD infection was significantly associated with age [age 25~35 years VS age <25 years (aOR = 1.520, 95%CI:1.062, 2.176); age≥35 years vs.age<25 years((aOR = 2.531,95%CI: 1.609,3.983)], and drug use (aOR = 1.946, 95%CI: 1.346,2.815). MSM with old age and using club drugs were more likely to have the HIV/STD infection.

**Table 5:** Factors associated with HIV/STD infection among MSM in Changsha, China.

	β	Wald *x* ^2^	P-value	aOR	95% CI	
					lower	upper
Age group						
<25(R)						
25~	0.419	5.228	0.022	1.520	1.062	2.176
≥35	0.929	16.126	0.000	2.531	1.609	3.983
Drug use						
No(R)						
Yes	0.666	12.511	0.000	1.946	1.346	2.815
constant	-1.591	115.128	0.000	0.204		

Note: R:reference group;

## Discussion

The current study revealed that 21.4% of MSM had a history of drug use during the past six months, similar to the study in Shenyang[22]and Washington DC [23]. Among Changsha MSM, poppers were the most commonly used single drug and poly-drug combinations, compared with methamphetamine in other six Chinese cities[24], ecstasy in Shanghai[25], and poppers in Shenyang[22]. This might be because poppers relax rectal muscles to reduce dyspareunia, and are cheaper and more easily accessible in the market and on the internet in China. In addition, local developed entertainment industries and popular club culture might be contributing factors.

The results also revealed that MSM with the low level of education, younger age, seeking partners through the internet or bars were more likely to use club drugs.This suggests that drug use surveillance and intervention targeted this group of MSM are of increased importance [26].

Although there were no significant differences in unprotected sexual intercourse and condom use between drug users and non-users, more drug users reported the experience of having sex intercourse with foreigner, multiple sex partners, and group sex. Our results had also shown club drug use was correlated with HIV/STD infection and might increase the risk of HIV/STD transmission, which was in keeping with previous findings among MSM in developed countries[27,28].Although it is difficult to judge whether the links reported here are cause-effect, these results still hint the presence of the relationship among HIV/STD infection, sexual risk behaviors, and drug use in Chinese MSM. Consistent with this, a multicenter AIDS cohort study also found that individuals that use poppers and methamphetamine are 2.10- and 1.46-times more likely to be HIV-positive[29]. Because most drug users in the current study used poppers alone or in combination with other drugs, the association between drug use and HIV/STD infections mainly reflects the effects of poppers. These results confirm that the use of poppers could increase the risk of infection with HIV/STD among Chinese MSM. Therefore, interventions for HIV/STD among Changsha MSM should consider the management and surveillance of club drugs, particularly popper use, and develop novel targeted HIV prevention strategies for drug-using MSM.

### Study Limitations

There are some limitations for the current study that should be considered. The participants were recruited by a convenient sample, although attempts were made to reduce selection bias using multiple recruitment techniques, the younger, single MSM were more likely attracted in the study. The cross-sectional study design also makes it difficult to make a causal inferences between the club drug use and HIV/STI. Finally, much sensitive private self-report information such as the number of sex partners and condom use, might have information bias.

Despite the aforementioned limitations, the current study is one of a small number of reports that assess the prevalence of club drug use and the association between club drug use and HIV/STD infections in China. The researching information will be helpful to develop effective behavioral interventions to reduce club drug use and the sexual transmission of HIV in China.

## Supporting Information

S1 File Complete dataset. (XLS)
